# A call for a coherent One Health strategy for the surveillance of climate-sensitive infectious diseases in the Canadian Arctic and subarctic regions

**DOI:** 10.1186/s42522-024-00117-5

**Published:** 2024-12-01

**Authors:** Laurence Daigle, Charlotte Nury, Léa Delesalle, Carol-Anne Villeneuve, Juliette Colinas, Patrick A. Leighton, Hélène Carabin, Kate Zinszer, Sean Hillier, Emily Jenkins, Cécile Aenishaenslin

**Affiliations:** 1 Faculté de médecine vétérinaire, Université de Montréal, Saint-Hyacinthe, Québec Canada; 2 Groupe de recherche en épidémiologie des zoonoses et santé publique (GREZOSP), Faculté de médecine vétérinaire, Université de Montréal, Saint-Hyacinthe, Québec Canada; 3 Centre de recherche en santé publique de l’Université de Montréal et du CIUSSS du Centre-Sud-de-l’île-de-Montréal, Montréal, Québec Canada; 4 Université de Montréal, Montréal, Québec Canada; 5 École de santé publique, Université de Montréal, Montréal, Québec Canada; 6 School of Health Policy & Management, York University, Toronto, Ontario Canada; 7 Department of Veterinary Microbiology, Western College of Veterinary Medicine, University of Saskatoon, Saskatoon, Saskatchewan Canada

**Keywords:** One health, Climate change, Climate-sensitive, Infectious disease, Arctic, Canada, Surveillance, Monitoring

## Abstract

**Introduction:**

The increased burden of climate-sensitive infectious diseases (CSIDs) within the circumpolar region, one of the many impacts of climate change, is impacting human, animal and ecosystem health. An integrated One Health approach to surveillance of CSIDs has been promoted by the scientific community as a prerequisite to enhance preparedness and response. Up to now, little is known about how the One Health approach has been implemented in surveillance systems for CSIDs in the Arctic and surrounding regions.

**Objectives:**

The objectives of this study were to map surveillance activities currently implemented in the Canadian Arctic and subarctic for the 16 CSID identified by the Arctic Council, to describe how One Health has been operationalized in these activities, and to explore the integration and leadership of Indigenous partners in current surveillance systems.

**Method:**

We performed the mapping in three steps: a rapid review of the scientific literature, a review of the grey literature and an online questionnaire sent to key stakeholders involved in CSID surveillance in the Canadian Arctic and subarctic regions.

**Results and conclusions:**

We identified 37 scientific peer-reviewed and 58 grey literature records. We mapped (1) surveillance of mandatory notifiable diseases at the federal, provincial or territorial levels not specific to the Arctic and subarctic regions, and (2) non-mandatory surveillance programs specific to the Arctic and subarctic regions. We described programs targeting either a single disease, human populations or wildlife. In most programs, there was no explicit mention of the integration of the One Health approach, and little information was available on collaboration efforts between sectors. Programs involved Indigenous communities at various levels, ranging from very low communication to community members, to high involvement and leadership in program management. Improvement in current CSID surveillance activities in Canada should include enhancing information accessibility, ensuring geographic representation, fostering sustainability in implementation of One Health strategies, and stronger involvement of Indigenous communities in the leadership of surveillance systems. An internationally harmonised approach across the Arctic and subarctic regions for all CSIDs has the potential to unify circumpolar surveillance efforts, save resources, and ultimately better inform public health authorities on the actions to prioritize in the context of climate change.

**Supplementary Information:**

The online version contains supplementary material available at 10.1186/s42522-024-00117-5.

## Introduction

The Arctic is inhabited by over 7 million people, including many Indigenous communities [1] from more than 40 nations [2]. In the Americas, the Inuit, Cree and Innu peoples, and in Eurasia, the Saami, Nenets and Evenk peoples, are amongst the main Indigenous peoples living in the Arctic and subarctic regions. In these regions, global warming is occurring nearly four times faster when compared to the rest of the world [3] which raises concerns for human, animal, plant and ecosystem health; one of these concern is the emergence of diseases [4, 5]. Indeed, climate change is modifying the ecology and spread of several infectious diseases, referred to as climate-sensitive infectious diseases (CSIDs) [6, 7]. The climate, along with its associated weather patterns, influence pathogens – namely, viruses, bacteria, fungi and parasites – whose transmission cycles involve animals, vectors or environmental compartments. The mechanisms underlying these disturbances include changes in pathogen survival and development rates outside the host, shifts in geographic range and abundance of reservoir animal species and disease vectors, and changes in transmission due to more frequent extreme climate events such as drought and heavy precipitation events [8]. In northern regions, research also suggests that the thawing of permafrost may expose populations and wildlife to pathogens preserved in ice, some of which may have been eradicated or forgotten, posing a potential challenge to modern immune systems not previously exposed to these agents [9].

The Arctic Council, a leading intergovernmental forum on Arctic governance, established an International Circumpolar Working Group (ICWG) in 2011 “to assess the potential emergence and health impact of climate-sensitive infectious diseases in northern human and animal populations, and to identify activities that may minimize the risks of disease emergence” [10]. This group identified 16 CSIDs of concern for human and animal health, caused by pathogens including viruses (hantaviruses, rabies virus, tick-borne encephalitis viruses, West Nile virus), bacteria (*Brucella* spp., *Leptospira* spp., *Clostridium botulinum*, *Francisella tularensis*, *Borrelia burgdorferi*, *Bacillus anthracis*, *Coxiella burnetti*) and parasites (*Toxoplasma gondii*, *Trichinella* spp., *Echinococcus* spp., *Giardia* spp., *Cryptosporidium* spp.) [10]. Except for botulism, which can survive in the form of spores in the environment, all these diseases are zoonotic agents and have wildlife reservoirs [11]. Consequently, the development of integrated surveillance systems following a One Health (OH) approach has been considered essential to better understand changing risks for humans and animals, and strengthen preparedness and response [7, 10, 12].

The Quadripartite Alliance defines OH as an “integrated, unifying approach that aims to sustainably balance and optimize the health of people, animals, and ecosystems, […] recognizing [that] the health of humans, domestic and wild animals, plants, and the wider environment (including ecosystems) are closely linked and inter-dependent” [13]. One Health surveillance systems thus require collaboration between at least two sectors (human health, animal health, plant health, environment) at different steps along the process, including planning, data collection, analysis, interpretation and communication phases [14]. The OH approach also embraces Indigenous Knowledge and ways of being regarding the intimate linkages between health, wellness and nature, and building upon this knowledge [15].

A growing number of research projects on CSIDs in the Arctic and subarctic regions have been developed and funded in recent years (see ArcticNet, 2021, amongst others) and apply the OH approach in partnership with Indigenous communities [16]. For example, the Nordic Centre of Excellence named CLINF is a research center, which is made up of an interdisciplinary research team collaborating with more than 150 local organizations in the Arctic region [17, 18]. The main objectives of this group are to identify and investigate the effects of climate change on human and animal infectious diseases from western Greenland to eastern Siberia in a One Health perspective, in order to provide accessible data and tools for communities and decision-makers. However, surveillance capacities for CSIDs in the Canadian Arctic and subarctic regions have never yet been formally assessed. It remains unclear to what extent the OH approach has been integrated into surveillance systems for CSIDs in these regions and whether Indigenous Knowledge is taken into consideration.

The objectives of this study were to map current surveillance activities and programs on CSIDs in the Canadian Arctic or subarctic regions, to characterize the actual implementation of the OH approach, and to explore the integration and the leadership of Indigenous partners in current surveillance systems. This article presents the main findings and lessons learned from this mapping, emphasizing the need for a coherent OH surveillance strategy for CSIDs led by the Indigenous communities affected by these diseases.

## Method

Current surveillance activities were mapped using three steps: a rapid review of the scientific literature, a review of the grey literature and an online questionnaire sent to key stakeholders involved in CSID surveillance in the Canadian Arctic and subarctic regions. The CSIDs included were the 16 diseases identified by the ICWG, as listed above [10].

**Fig. 1:**
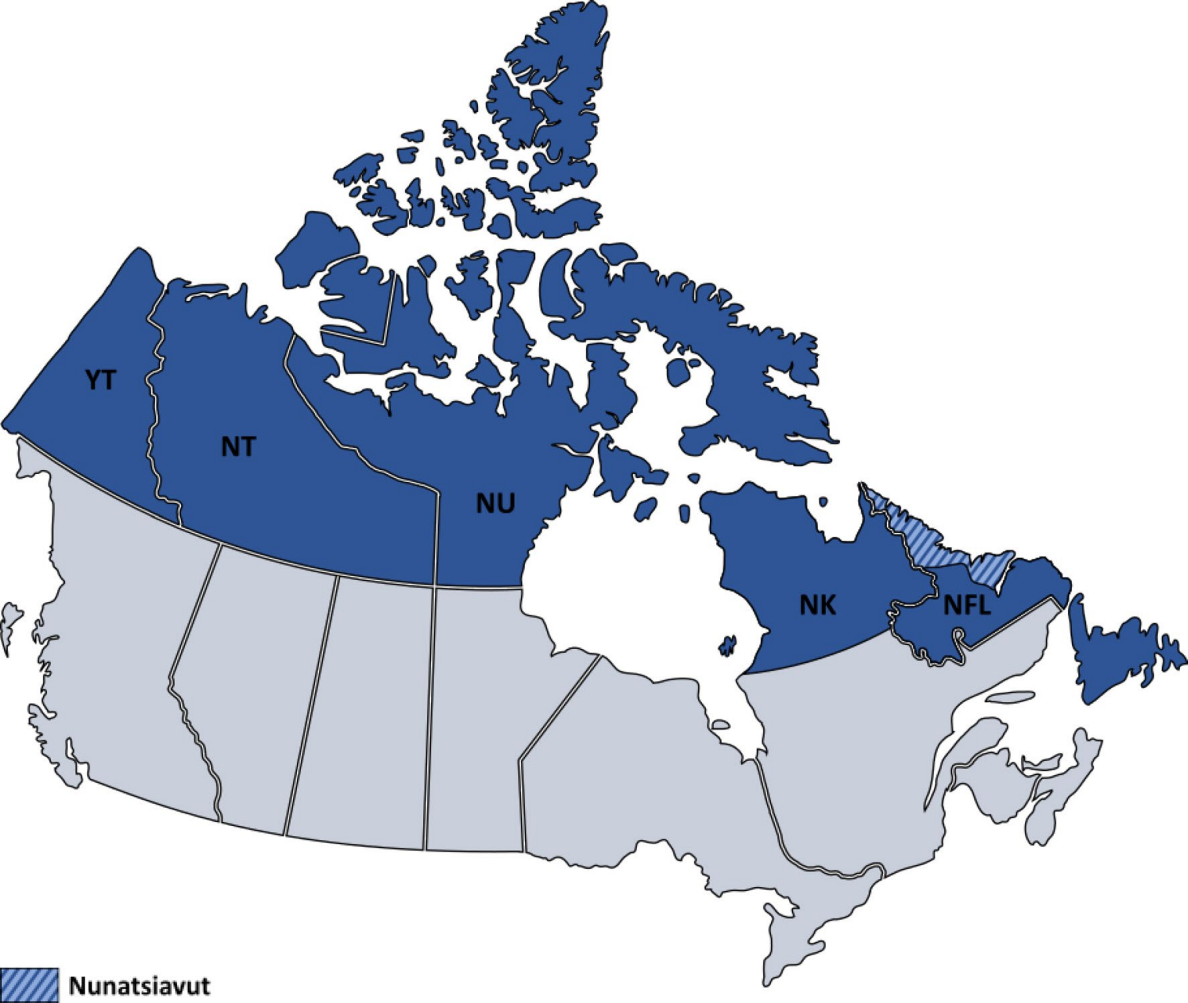
Map of the selected study area. Legend: The selected study area covers lands acknowledged by the Arctic Council as part of the Canadian Arctic and subarctic regions. It includes the following territories and provinces, in blue: Yukon (YT), Northwest Territories (NT), Nunavut (NU), a part of the Québec (Nunavik; NK) and Newfoundland and Labrador (NFL), including Nunatsiavut (hatched area)

The selected study area covers lands acknowledged by the Arctic Council as part of the Canadian Arctic and subarctic regions and is presented in Fig. 1, including Yukon (YT), Northwest Territories (NT), Nunavut (NU), Nunavik (NK; Québec) and Newfoundland and Labrador (NFL), including Nunatsiavut.

Surveillance activities were defined as any multi-year activity or program involving systematic data collection, integration and analysis, and timely dissemination of information to inform public or animal health decisions [19].

### Scientific and grey literature review

The rapid scientific literature review was conducted in PubMed and Web of Science (including Core Collection, Biological Abstracts, Medline), using keywords associated with four themes: CSID, Canadian Arctic and subarctic regions, surveillance and One Health (Table 1 and Appendix 1). A general framework for conducting rapid literature reviews as outlined by the 2020 Cochrane Rapid Reviews guidance document was followed with modifications [20]. The review included the following steps: (1) database search, (2) importation of the records into an Excel spreadsheet, (3) removal of duplicates, and (4) rapid screening of titles and abstracts for eligibility (one reviewer per record). The grey literature review included a search within all websites of Canadian government entities (federal, provincial and territorial) and of Indigenous communities, research compendiums and topic-focused grey literature databases (such as Arctic Science and Technology Information System (ASTIS) and Circumpolar Health Bibliographic Database (CHBD)). Google and Google Scholar search engines broadened the scope of the review. For the grey literature, we adapted the search strategy to the constraints of each database, sometimes resorting to single-word or manual search when necessary. An initial search was conducted between May and December 2021, followed by a final search between April and November 2023 to identify new, relevant papers, reports and information on websites. The eligibility of both grey and scientific evidence was assessed independently by three reviewers (LDe, CN and LDa). Records were selected based on the following inclusion criteria: English or French languages, reliability of the source of information for grey literature (websites, reports or online documents from governments or research organizations), online accessibility, relevance to the subject, geographic range limited to Arctic and subarctic regions of Canada and activities ongoing since 2000 or that have lasted at least one year. Records were excluded according to the following exclusion criteria: not a multi-year project, no mention of a surveillance program, wrong geographic area and does not include any of the 16 targeted CSIDs.

### Stakeholder questionnaire

To complete information on surveillance activities identified in the scientific and grey literature, and to collect information on other surveillance activities that we may have missed, we contacted key stakeholders involved in infectious disease surveillance within the Canadian Arctic and subarctic regions and asked them to complete a short questionnaire about these surveillance systems. We used two means of recruitment: (1) we contacted key stakeholders identified by consulting websites of Arctic research networks, Indigenous institutions and government organizations through the scientific and grey literature (i.e. project or program leaders and corresponding authors); (2) at the end of the questionnaire, participants were invited to name other stakeholders involved in surveillance activities in the Canadian Arctic and subarctic regions. This resulted in the identification of 49 stakeholders who were invited by email to participate in an online questionnaire designed using the LimeSurvey Professional software [21]. The questionnaire was composed of five questions (available in Appendix 2) to collect additional information about ongoing or past surveillance activities or programs. It was conducted between May and December 2021.

### Data collection and analysis

We systematically extracted information on the CSID surveillance systems in the Canadian Arctic and subarctic regions identified with the three steps described above (additional details are available in Appendix 3). This included: program name, lead organization, targeted diseases and pathogens, type of data collection (opportunistic, i.e., identified through other activities, active or passive), geographic areas covered, stakeholders involved, targeted OH sectors (human health, animal health, plant health, food safety, wildlife and environmental health) and Indigenous participation. To characterize the implementation of OH in surveillance activities based on the extracted information, we used the following definition of OH integration into disease surveillance: “the collaborative efforts […] between at least two sectors (amongst human health, animal health, plant health, food safety, wildlife and environmental health) at any stage of the surveillance process […]” [14]. To explore the involvement of Indigenous partners in current surveillance systems, we searched for evidence of participation and/or leadership from Indigenous organizations. We characterized the participation as follow: (1) the program was explicitly managed by an Indigenous group or organization (characterized as “lead”), (2) specific participation was mentioned (characterized as “collaborators”), and (3) there was not enough information on the type of involvement (characterized as “unclear”). The information from the three steps detailed above was combined to map the state of current CSID surveillance in the Canadian Arctic and subarctic regions.

## Results

### Search results

**Fig. 2:**
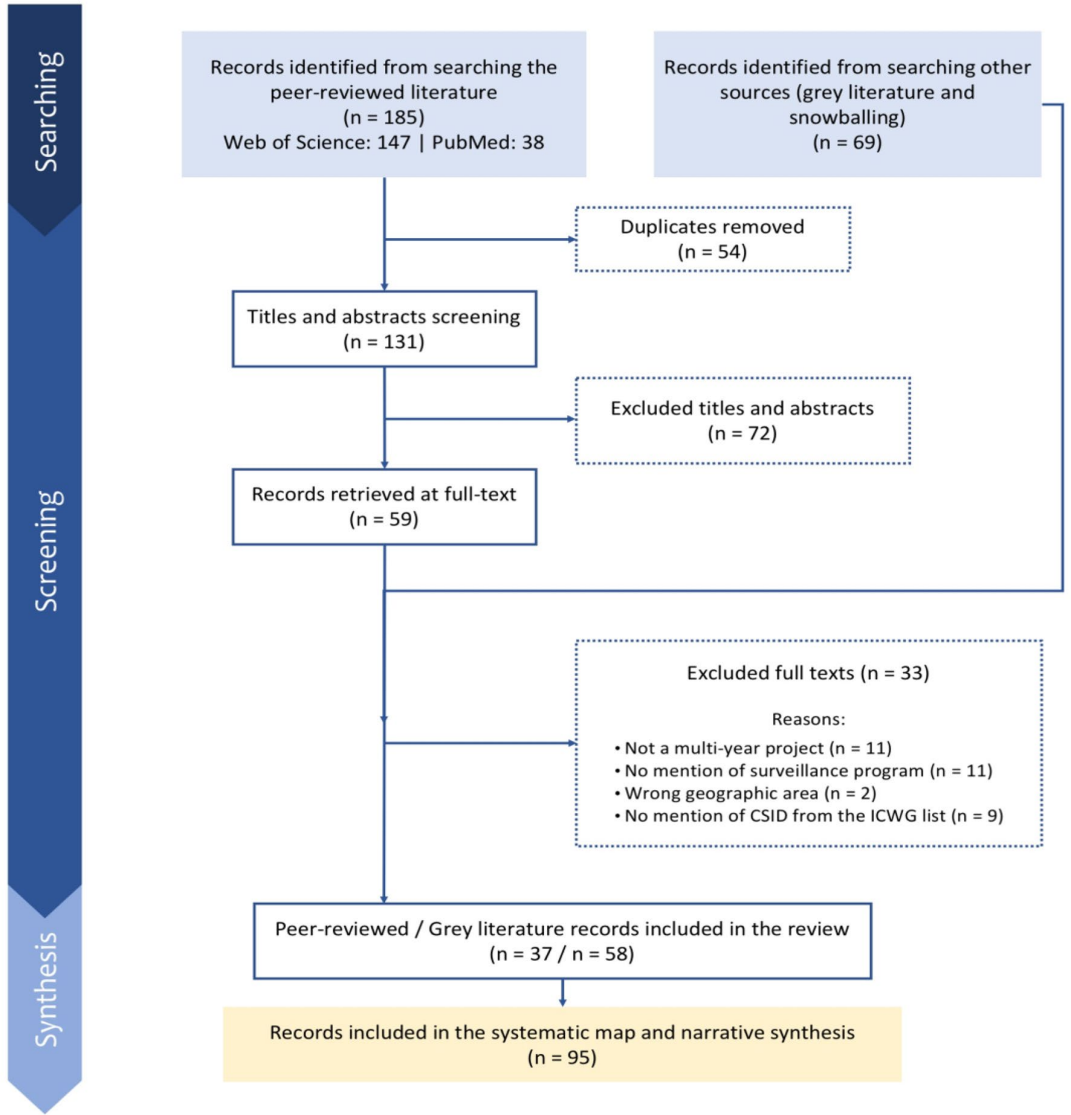
Flow diagram of the mapping for current CSID surveillance activities. Legend: This flow diagram presents the number of results obtained at each step of the search. Excluded articles did not meet the inclusion criteria. A record does not necessarily refer to a surveillance program or network (e.g. one program may ask for multiple research permits over the years, hence generating multiple records in a compendium). Once extracted, records were analyzed and grouped together by stakeholders and targeted diseases to have an accurate view of the current surveillance programs

A flow diagram synthesizing search results from both the grey and scientific literature is presented in Fig. 2. In total, 37 scientific peer-reviewed and 58 grey literature records were identified (n, total = 95). Record exclusion was mainly due to not being a multi-year project (*n* = 11), having no mention of a surveillance program (*n* = 11), lacking reference to CSIDs from the ICWG list (*n* = 9), and being in the wrong geographic area (*n* = 2). Out of the 49 key stakeholders invited to complete the online questionnaire, only 10 responded with enough information to be included in our mapping results. All of the respondents were representatives from federal or provincial government or were part of academic research groups.

All Arctic and subarctic regions targeted in this study were covered by at least one surveillance program. This coverage included nationwide passive surveillance activities for mandatory reportable diseases and non-mandatory surveillance programs. The Northwest Territories and Nunavut were the regions with the highest coverage from CSID surveillance programs, followed by the Nunavik region (Québec). Yukon and Newfoundland and Labrador were the least covered regions.

The surveillance activities included in the analysis can be divided into two main sections, that are (1) surveillance of mandatory notifiable diseases at the federal, provincial or territorial levels that are not specific to the Arctic and subarctic regions, and (2) non-mandatory surveillance programs specific to the Arctic and subarctic regions. Both sections are described below.

### Mandatory notifiable diseases at the federal, provincial or territorial levels that are not specific to the Arctic and subarctic regions

The term “notifiable disease” encompasses all diseases that require immediate and/or annual notification. Cases of these diseases in humans or animals are reported by physicians, laboratories and/or veterinarians in each province and to the federal authorities, contributing to the surveillance system. Amongst the CSIDs included in this study, six out of 16 were notifiable to public health authorities in Canada at the time of publication, for both humans and animals: anthrax, botulism, brucellosis, rabies, tularemia, and West Nile fever [22–24]. Four out of 16 CSIDs were notifiable for animals only (echinococcosis, Q fever, toxoplasmosis, and trichinellosis), and four were notifiable for humans only (cryptosporidiosis, giardiasis, hantavirus pulmonary syndrome, and Lyme disease). Leptospirosis and tick-borne encephalitis were the only two CSIDs without obligation of notification to the federal authorities. Table 2 summarizes the different categories related to federal reportable diseases.

Each province and territory also maintains its own notifiable or reportable disease list, indicating additional diseases for surveillance in addition to federally reportable diseases. These lists change over time, depending on the priorities of provinces and territories. For example, leptospirosis, which is not a reportable disease at the federal level, was notifiable in Newfoundland and Labrador, Ontario, and Québec at the time of the study [22].

All these surveillance activities are led by provincial, territorial, and federal public authorities. Professionals practicing in the Arctic and subarctic regions at local institutions are required to report these diseases as in other Canadian regions, but we found no clear documented evidence of participation of local Indigenous communities in the leadership of these programs.

### Non-mandatory surveillance programs specific to the Arctic and subarctic regions

In addition to the notifiable reporting systems of CSIDs within federal, provincial, or territorial programs, surveillance programs led by other organizations and targeting one or more CSIDs were identified. These surveillance programs can be further classified into three sub-categories: (A) programs targeting a single disease (including screening programs), (B) surveillance programs targeting human populations, or (C) surveillance programs targeting wildlife (monitoring one animal species or overall wildlife health). The first two categories are presented in Table 3.

A) **Programs targeting a single disease.** Three programs were identified in this category and targeted two CSIDs: trichinella and West Nile virus. The two programs targeting trichinella are programs led by Indigenous communities to screen walrus meat submitted by Indigenous hunters for the presence of pathogens [25–28]. The Nunavik Trichinellosis Prevention Program has been ongoing for over 20 years, while the Nunavut Trichinella Detection Program is a more recent initiative (2017). The third program targeted West Nile virus across Canada through a combination of active and passive surveillance activities. This program is led by the Canadian Wildlife Health Cooperative (CWHC), an organization focused on wildlife conservation and management in Canada. In this monitoring strategy, data were collected through human blood and organ donations, testing birds or horses that were sick or dead, examining mosquito pools, and screening humans exhibiting symptoms compatible with West Nile fever [29, 30]. With this program, we found no clear evidence of active involvement of Indigenous communities.
B) **Surveillance programs targeting human populations.** Two programs were classified in this category (Table 3, B) [27, 31]. The National Inuit Health Surveys (Qanuippitaa?) are conducted through partnerships by academic researchers and Inuit organizations. Since 2018, it has been fully led by Inuit communities. This program collects data on exposure to toxoplasmosis, rabies, trichinellosis, and cryptosporidiosis. The program’s objective is to collect data to improve the health and well-being of Inuit, including health data on disease prevalence [32]. The Nunavik Toxoplasmosis Screening Program for pregnant women was implemented by academic researchers, local government, and Inuit organizations in response to cases reported in Nunavik in the 90s. Since 2004, this program has been part of the National Inuit Health Surveys.
C) **Surveillance programs targeting wildlife**. Programs included in category C were more complex and intertwined, often mergers of existing monitoring programs targeting wildlife species. Different organizations (e.g. academics, federal or provincial/territorial governments, local groups, non-governmental organizations, etc.) were responsible for various surveillance functions, including funding, data collection, laboratory analysis, data analysis, interpretation and integration, and results dissemination. Additionally, some of these activities are embedded within broader research programs. Therefore, it was challenging to distinguish between different surveillance activities and the involved groups and institutions. The main characteristics are described below.

At least thirteen CSIDs were covered in these different programs (13/16): anthrax, brucellosis, cryptosporidiosis, echinococcosis, giardiasis, hantavirus pulmonary syndrome, leptospirosis, Lyme disease, rabies, toxoplasmosis, trichinellosis, tularemia, and West Nile fever. Based on the information available on these programs, they involved Indigenous communities at various levels, either mostly collaborators or leads of certain activities.

The most frequently monitored species in this category were **Caribou** [33–44], **Wood bison** [34, 40–43, 45–57], **Beluga** [34, 40–43, 58–60], **Polar bear** [61–65], **Ringed and bearded seal** [34, 41–43, 53, 66–70] and **Muskox** [71–78]. Some of those species were classified, at the time of publication, as species of concern or priority species. Indeed, in Canada, Barren-ground Caribou, Boreal Caribou, Peary Caribou, and Wood Bison are amongst the six federally, provincially, and territorially shared priority species identified, and they are located in Arctic and subarctic regions. For those species, federal, provincial, and territorial governments are collaborating to develop recovery strategies, either following the Pan-Canadian Approach or focusing on Priority Species. With this approach, the government is funding surveillance activities intending to protect these species.

Other surveillance activities in this category targeted more than one animal species [34, 40, 42, 49, 52, 53, 56, 66, 70, 79–94]. One example is the “Community-based monitoring program of wildlife health”, coordinated by a research group based at the University of Calgary, Canada [43, 77, 89, 93, 95–98]. This ongoing program started in 2003 in the Northwest Territories and was later extended to Nunavut. As a community-based program, it evolved over time to incorporate Indigenous Knowledge, and some monitoring activities were led by local Indigenous groups. It also includes many organizations and groups, such as academia, regional and local groups, non-governmental organizations and government. Over the years, many CSIDs were covered at various levels of investigation in this program, including brucellosis, echinococcosis, toxoplasmosis, and West Nile fever. Other suspected CSIDs not included in the list were also investigated, as orf and erysipelas.

### Implementation of One Health

We found no explicit mention of the integration of the OH approach in the retrieved activities, except for one example which can be included in category C, namely *The Canadian Arctic One Health Network* [16]. It is a network of researchers and community partners focused on monitoring, modeling, and mitigating various One Health threats across the changing Canadian North. Still, some programs can be considered as implementing OH surveillance, as per Bordier et al.’s definition. Surveillance activities integrating two to four sectors were identified, with most programs involving collaborating efforts, at some level, between two sectors. The nationwide surveillance of West Nile virus (in mosquito pools, wild birds, horses, and people) serves as an example of data integration across various sectors, although this surveillance system was not specific to the Arctic and subarctic regions. This monitoring program is the result of a close collaboration between multiple provinces, territories and federal agencies, as well as non-governmental organizations (NGOs), to actively track cases of West Nile virus in wildlife (birds), domestic animals (horses), the environment (mosquito pools), and humans (hospital-reported cases) each summer.

The two OH sectors most frequently involved in CSID surveillance were ‘wildlife & environmental health’ and ‘food safety’. Category A (targeting one disease) was the category with the best coverage of OH sectors, including wildlife health, food safety, human health and domestic animal health. Programs in Category B (targeting human populations) included only the human health and food safety sectors. Programs in Category C (targeting wildlife) covered mostly wildlife health and food safety. The CWHC programs in this category also had a certain orientation towards human health. Little information was available on how collaboration efforts between sectors took place. For example, it was not possible to determine whether collaboration was limited to the joint interpretation of surveillance data from multiple sources (e.g., animals and food products) or involved co-development or co-management by multiple institutions.

## Discussion

This study is the first to systematically map and characterize the implementation of OH and the integration of Indigenous Knowledge and leadership in surveillance systems for CSIDs in the Canadian Arctic and subarctic regions. Results reveal that although some surveillance activities were found for almost all CSIDs prioritized by the Arctic Council, major gaps were identified regarding the equitable inclusion of regions across the Canadian Arctic and subarctic regions that are the most strongly affected by climate change, the implementation of a sustainable One Health approach, and the integration of a strong Indigenous leadership in existing initiatives.

### Geographic representation

Surveillance activities appeared to be unevenly distributed across the Northern region, with some territories and/or communities better represented than others. Indeed, the majority of the surveillance programs were located in the Northwest Territories and Nunavut, whereas Yukon and Newfoundland-Labrador were the least-covered areas. Other northern Indigenous communities in Canada, especially those in subarctic regions, are also suffering from climate change and emerging diseases and could benefit from a more inclusive CSID surveillance network.

### One Health implementation and sustainability

OH implementation was not explicitly mentioned in any surveillance documentation retrieved in this study. There was also no available information on the collaboration efforts between sectors, including co-planning, data collection, management and sharing, dissemination of findings, and communication of positive cases between agencies. Nevertheless, a partnership amongst academics, governments, NGOs and local groups is crucial for complex surveillance programs, and most of the programs included at least some representation of these groups. A more indepth evaluation of OH integration into CSID surveillance programs currently taking place in the Canadian Arctic and subarctic regions would be beneficial to obtain more accurate and comprehensive information before identifying and addressing gaps [14, 99].

Another important finding was that most of the non-mandatory surveillance activities were not long-term formal surveillance programs, but rather multi-year activities conducted by a research network or team, which can impede the sustainability of current CSID surveillance. This can be mainly observed in Category C (targeting wildlife), which includes programs that involved many organizations and various sources of funding. Although the inclusion of multiple partners in the surveillance of CSIDs can create synergies between surveillance and research, this approach can also be a threat to surveillance programs if their activities rely on research funds. Long-term surveillance programs are essential for the continuous monitoring and evaluation of CSIDs affecting humans, animals, and their shared environments and could provide the information needed to detect, prevent, and control these diseases [100]. The lack of adequate surveillance systems for CSIDs in the North has been noted by other authors [7, 101], as is the need for the development and implementation of sustainable CSID surveillance systems in the Arctic and subarctic regions [102]. Awuor and collaborators (2019) propose that this gap is partly due to the availability of sufficient data on the risks of a disease justifying stopping a program, or to a lack of financial resources [103]. However, as they pointed out, sustainable and continuous surveillance activities are critically needed to assess risks, fully understand the situation, and prepare for future emergences.

### Leadership of Indigenous communities

The findings of this study also underline an apparent lack of involvement from Indigenous communities in certain programs, despite all of these activities taking place on native lands. Detailed reporting of participatory processes is crucial for the co-production of knowledge in the Arctic [104]. It is also essential that Indigenous people be at the core of the management and design of CSID surveillance programs in the future [7, 101]. Equitable inclusion of diverse knowledge systems achieves better outcomes than a sectoral framework, particularly in the circumpolar region where the majority of the population is Indigenous [105, 106]. Integration of Indigenous Knowledge in research and surveillance of health threats for humans, animals, and ecosystems is also known to contribute meaningfully to adaptation to climate change [107, 108].

Despite these remaining challenges, some examples suggest positive changes over time toward stronger Indigenous leadership. For example, Inuit Tapiriit Kanatami (ITK) became the lead and developed the Qanuippitaa? National Inuit Health Survey (QNIHS) from 2021 onwards [31]. Before 2021, similar Indigenous health surveys were conducted through partnerships between researchers, Inuit organizations and local health authorities, but not led by Indigenous communities. Another example identified in this study is the “Community-based monitoring program of wildlife health”, which integrates local and Indigenous Knowledge with western ecological knowledge [95, 109]. Other existing good examples of Indigenous Knowledge integration in research and practices could serve as models to enhance current practices in CSID surveillance. A mapping of all community-based monitoring activities in the Arctic by the Sustaining Arctic Observing Networks led to the publication of the web Atlas Nunaliit in 2015 (http://www.arcticcbm.org) [110]. While not specifically targeting CSIDs, this work highlighted good practices for conducting monitoring activities in partnership with Indigenous communities. These practices include capacity building, co-production of knowledge, support for the construction of sustainable networks, development of accessible data management protocols, use of the collected data to inform decision-making, and ensuring the sustainability of the programs [110].

### The need for a coherent, Arctic- and subarctic-wide, One Health surveillance strategy for CSIDs, co-managed by the Indigenous communities

A key priority for future developments in CSID surveillance in the Canadian Arctic and subarctic regions is to identify and prioritize CSIDs of interest for the various regions and communities involved. To achieve this, we propose employing a participatory research approach involving Indigenous community representatives.

To ensure that information on surveillance activities is more comprehensible and accessible, there are existing examples of mapping activities related to climate change. For instance, *Canada in a Changing Climate: Advancing our Knowledge for Action* produced a geographical map documenting case stories related to adaptations and resilience for addressing climate change in Canada [111]. Another example is The Northern Caribou Canada project, which aims to track changes in Canadian Arctic and subarctic caribou populations, incorporating both traditional and scientific knowledge [112]. The Wekʼèezhìi Renewable Resources Board, led by the Tłı̨chǫ Nation and supported by the federal and territorial government, created this project. These are examples of tools used for CSID surveillance activities that make the information more accessible to all organizations and groups, and to the public, including Indigenous Peoples.

### Limitations of the study

This study has limitations. Firstly, during our research, we excluded surveillance programs for infectious diseases in the Arctic and subarctic regions that were not listed in the International Circumpolar Working Group, but which could also be considered as CSIDs. For instance, the International Circumpolar Surveillance system collects data on invasive bacterial diseases, but none are pathogens prioritized by the Arctic council [113–116]. Numerous authors have emphasized a possible increased burden for several infectious diseases not included in this study, including enteric infections [117], helminthic zoonoses [101, 118], *Erysipelothrix rhusiopathiae* [119], rickettsial diseases and mosquito-borne viruses, including California serogroup viruses [120, 121] in the Northern regions within the context of changing climate. Also, since the study, several other infectious agents that could be of concern have emerged, but were not included.

Secondly, we encountered considerable difficulty in accessing information on existing surveillance activities. Only a few formal surveillance programs were described in the grey and scientific literature, and their descriptions lacked detail. Since many of them involved diverse organizations (i.e. academia, federal and/or regional governments, non-governmental organizations, local groups, etc.), it was challenging to delineate their frontiers, due to the lack of information provided. This is why we decided not to list and count programs in Category C, as done with Categories A and B which were less challenging on this matter. The lack of geographic representation in certain Canadian Arctic and subarctic regions could be partly due to a representation bias, as some provinces maintain better records of activities (research compendium available at the NT and NU) compared to other provinces or territories, for which information on activities was more difficult to obtain. Information on CSID surveillance activities needs to become available and more accessible for Indigenous Peoples, decision-makers, and researchers to inform the development of a coherent OH surveillance strategy for CSIDs across the Canadian Arctic and subarctic regions. There is a crucial need for guidelines for better describing surveillance programs.

Lastly, the stakeholders questionnaire had a very low response proportion, and most respondents from provincial or federal authorities were not directly involved in surveillance programs. This led to limited access to information and a limited depth of understanding of the programs. Therefore, due to the limited access to information about programs, the classification of monitoring activities in Table 3 may not be the most adequate representation. Moreover, surveillance activities were challenging to characterize: there was no official name or topic mentioned, pathogens searched were sometimes not clearly defined, and roles and parties involved in the surveillance were often not clearly identified, varying over time in longitudinal projects.

**Table 1:** Key concepts and keywords for this systematic map

Categories	Subcategories	Search terms used^a^
Surveillance activities	Surveillance	Surveillance, track, monitor
	Activities	Activity, network, project, program
CSID	Climate-sensitive	Climate, climate-sensitive
	Infectious diseases	Bacteria, disease, illness, infection, pathogen, virus
	Bacterial CSID	*Bacillus anthracis* (Anthrax), *Borrelia burgdorferi* (Lyme disease), *Brucella* spp. (brucellosis), *Clostridium botulinum* (botulism), *Coxiella burnetti* (Q Fever), *Francisella tularensis* (tularemia), *Leptospira* spp. (leptospirosis)
	Parasitic CSID	*Cryptosporidium* spp. (cryptosporidiosis), *Echinococcus* spp. (echinococcosis), *Giardia* spp. (giardiasis), *Toxoplasma gondii* (toxoplasmosis), *Trichinella* spp. (trichinellosis)
	Viral CSID	hantaviruses, rabies, tick-borne encephalitis viruses, West Nile virus
Canadian Arctic and subarctic^b^ regions	Territories	Canadian Arctic, Canadian subarctic, circumpolar, Labrador, Northwest Territories, Nunavut, Yukon
	Northern parts of some provinces	Inuvialuit, Nunatsiavut, Nunatukavut, Nunavik
One Health		One health, ecohealth

^a^Search terms were sometimes truncated for better inclusivity of plurals and derivatives

^b^The subarctic region includes regions located North of several provinces in Canada, which has often been overlooked in Canadian northern research [122]. The Arctic and subarctic regions of Canada include all regions situated North of the southern limit of discontinuous permafrost [123]

**Table 2:** CSIDs that are notifiable at the federal level, for terrestrial animals [22, 23] and humans [24].^a^

Diseases	Animal	Human (date the disease became notifiable)
Anthrax	X^R^	X^CPS^ (2002)
Botulism	X^AN^	X^OC^ (1933)
Brucellosis	X^R^	X^OC^ (1928)
Cryptosporidiosis	-	X^OC^ (2000)
Echinococcosis	X^AN^	-
Giardiasis	-	X^OC^ (1983)
Hantavirus pulmonary syndrome	-	X^OC^ (2000)
Leptospirosis	-	-
Lyme disease	-	X^CPS^ (2009)
Q fever	X^AN^	-
Rabies	X^R^	X^OC^ (1927)
Tick-borne encephalitis	-	-
Toxoplasmosis	X^AN^	-
Trichinellosis	X^R^	-
Tularemia	X^AN^	X^OC^ (2002)
West Nile fever	X^IN^	X^CPS^ (2003)

R: Reportable diseases – “Pet owners, veterinarians, and labs must report suspected cases of federally reportable diseases in Canada.”

AN: Annually notifiable diseases – “Diseases recorded in Canada for the annual report to the World Organisation for Animal Health.”

IN: Immediately notifiable diseases – “Diseases exotic to Canada with no control or eradication programs.”

CPS: “Confirmed, probable and suspect cases of disease should be notified.”

OC: “Only confirmed cases of disease should be notified.”

^a^Additional Provincial/Territorial reportable diseases may be determined by the province and territories, but they include de facto all federally reportable diseases. For the date that the disease became notifiable, some diseases had periods of time that they were notifiable followed by an interruption, before this date

**Table 3:** Overview of programs for CSIDs in the Canadian Arctic and subarctic regions

A. Programs targeting one disease (including screening programs)										
Description	Period	Disease	Type of data collection	Geographic Area^a^	Stakeholders	Sectors under surveillance	Indigenous involvement	Grey litterature	Scientific litterature	Consultation with key stakeholders
**Nunavik Trichinellosis Prevention Program** *Community-based public health screening program for hunter-submitted walrus meat.*	From 1992, and still ongoing	Trichinellosis	Passive	NK	Regional/Local	Wildlife health, Food safety	Lead	[25, 27, 28]	[124–126]	Yes
**Nunavut Trichinella detection program** *Local public health screening program for hunter-submitted walrus (and polar bear) meat.*	From 2017, and still ongoing	Trichinellosis	Passive	NU	Regional/Local, Territorial	Wildlife health, Food safety	Lead	[26]		
**West Nile virus Surveillance** *Pan-species federally coordinated surveillance program.*	Start date unclear, and still ongoing	West Nile virus	Active and passive	Canada	Federal, Provincial/ Territorial, Regional/Local, NGO	Human health, Wildlife health, Domestic animal health	Unclear	[29, 30, 127]		

**B. Surveillance programs targeting human populations**										
Description	Period	Disease	Type of data collection	Geographic Area^a^	Stakeholders	Sectors under surveillance	Indigenous involvement	Grey litterature	Scientific litterature	Consultation with key stakeholders
**Qanuippitaa? National Inuit Health Survey (QNIHS)** *Recurrent population health survey of Inuit people across Canada.*	From 2004, and still ongoing.	Toxoplasmosis, rabies, trichinellosis, cryptosporidiosis, Unknown (probable CSIDs)	Active	NU, NK, NFL, NT	Territorial, Provincial, Regional/Local	Human health, Food safety	Lead (since 2018)	[27, 31]	[32]	
**Nunavik Toxoplasmosis Screening Program for pregnant women** *Public health screening program for pregnant women in Nunavik.*	From 1994, and still ongoing	Toxoplasmosis	Active	NK	Regional/Local, Provincial	Human health, Food safety	Lead (since 2018)	[27]	[128, 129]	Yes

^a^ NFL = Newfoundland and Labrador | NK = Nunavik | NU = Nunavut | NT = Northwest Territories

## Conclusion

The scientific community widely acknowledges the importance of implementing and maintaining CSID surveillance in the Arctic and subarctic. Substantial investments in surveillance programs aimed at detecting disease emergence events are urgently needed, distinguishing newly detected diseases from emerging diseases, establishing baseline rates for endemic diseases susceptible to transmission in a warming North, and assessing the effectiveness of awareness and control programs in reducing transmission and enhancing biosecurity. Our study highlights the need for improved access to information about ongoing surveillance activities in the circumpolar region. In evaluating the gaps in the current surveillance activities on CSIDs in the Canadian Arctic and subarctic regions, there is a critical need for a coherent, Arctic- and subarctic-wide, One Health surveillance strategy for CSIDs, co-managed by Indigenous communities. This strategy requires a renewed effort in prioritizing needs and risks associated with CSIDs in the Canadian Arctic and subarctic regions. It aims to ensure more equitable distribution of surveillance activities across all parts of the Canadian Arctic and subarctic regions, establish sustainable programs over time, foster OH collaboration amongst the involved sectors, and, last but not least, ensure that Indigenous people are at the core of the management and design of these CSID surveillance programs. An internationally harmonised approach across the Arctic and subarctic regions for all CSIDs has the potential to unify circumpolar surveillance efforts and ultimately better inform public health authorities on the actions to prioritize in the context of climate change [18].

## Electronic supplementary material

Below is the link to the electronic supplementary material.

Supplementary Material 1

Supplementary Material 2

Supplementary Material 3

## Data Availability

The data that support the findings of this study are available from the corresponding author upon reasonable request.

## Abbreviations

ASTIS: Arctic Science and Technology Information System
CHBD: Circumpolar Health Bibliographic Database
CSID: Climate-sensitive infectious disease
CWHC: Canadian Wildlife Health Cooperative
ICWG: International Circumpolar Working Group
ITK: Inuit Tapiriit Kanatami
NFL: Newfoundland and Labrador
NGO: Non-governmental organization
NK: Nunavik
NT: Northwest Territories
NU: Nunavut
OH: One Health
QNIHS: Qanuippitaa? National Inuit Health Survey
WNV: West Nile virus
YT: Yukon

## References

[1] Jungsberg L, Turunen E, Heleniak T, Wang S, Ramage J, Roto J. Atlas of population, society and economy in the Arctic [Internet]. Nordregio. 2019 [cited 2020 Jul 22]. http://urn.kb.se/resolve?urn=urn:nbn:se:norden:org:diva-5711

[2] Dallmann WK. Indigenous peoples of the Arctic countries [Internet]. Norwegian Polar Institute; 2015. https://arctic-council.org/site/assets/files/4330/indig_peoples.png

[3] Rantanen M, Karpechko AY, Lipponen A, Nordling K, Hyvärinen O, Ruosteenoja K, et al. The Arctic has warmed nearly four times faster than the globe since 1979. Commun Earth Environ. 2022;3(1):1–10.

[4] Parkinson AJ, Butler JC. Potential impacts of climate change on infectious diseases in the Arctic. Int J Circumpolar Health. 2005;64:478–86. 16440610 10.3402/ijch.v64i5.18029

[5] Parkinson AJ, Evengård B. Climate change, its impact on human health in the Arctic and the public health response to threats of emerging infectious diseases. Global Health Action. 2009;2:2075. 10.3402/gha.v2i0.2075 PMC2799221 20052420

[6] Hedlund C, Blomstedt Y, Schumann B. Association of climatic factors with infectious diseases in the Arctic and subarctic region – a systematic review. Global Health Action. 2014;7:24161. 24990685 10.3402/gha.v7.24161 PMC4079933

[7] Dudley JP, Hoberg EP, Jenkins EJ, Parkinson AJ. Climate change in the North American Arctic: a one health perspective. EcoHealth. 2015;12:713–25. 10.1007/s10393-015-1036-1 26070525

[8] El-Sayed A, Kamel M. Climatic changes and their role in emergence and re-emergence of diseases. Environ Sci Pollut Res Int. 2020;27(18):22336–52. 32347486 10.1007/s11356-020-08896-w PMC7187803

[9] Waits A, Emelyanova A, Oksanen A, Abass K, Rautio A. Human infectious diseases and the changing climate in the Arctic. Environ Int. 2018;121:703–13. 30317100 10.1016/j.envint.2018.09.042

[10] Parkinson AJ, Evengard B, Semenza JC, Ogden N, Børresen ML, Berner J, et al. Climate change and infectious diseases in the Arctic: establishment of a circumpolar working group. Int J Circumpolar Health. 2014;73:25163. 25317383 10.3402/ijch.v73.25163 PMC4185088

[11] Harper SL, Edge VL, Ford J, Willox AC, Wood M, McEwen SA, et al. Climate-sensitive health priorities in Nunatsiavut, Canada. BMC Public Health. 2015;15:18. 26135309 10.1186/s12889-015-1874-3 PMC4489362

[12] Ruscio BA, Brubaker M, Glasser J, Hueston W, Hennessy TW. One health – A strategy for Resilience in a changing Arctic. Int J Circumpolar Health. 2015;74:27913. 26333722 10.3402/ijch.v74.27913 PMC4558275

[13] One Health High-Level Expert Panel (OHHLEP), Adisasmito WB, Almuhairi S, Behravesh CB, Bilivogui P, Bukachi SA et al. One Health: A new definition for a sustainable and healthy future. PLOS Pathogens. 2022;18(6):e1010537. 10.1371/journal.ppat.1010537 PMC9223325 35737670

[14] Bordier M, Uea-Anuwong T, Binot A, Hendrikx P, Goutard FL. Characteristics of one health surveillance systems: a systematic literature review. Prev Vet Med. 2020;181:104560. 10.1016/j.prevetmed.2018.10.005 30528937

[15] Hillier SA, Taleb A, Chaccour E, Aenishaenslin C. Examining the concept of one health for indigenous communities: a systematic review. One Health. 2021;12:100248. 33912647 10.1016/j.onehlt.2021.100248 PMC8066803

[16] ArcticNet AN. 2021 [cited 2023 Apr 24]. The Canadian Arctic one health network. https://arcticnet.ulaval.ca/fr/project/the-canadian-arctic-one-health-network/

[17] CLINF. CLINF. 2024 [cited 2024 Aug 9]. CLINF from Nuuk to Yakutsk. https://clinf.org/

[18] Evengård B, Destouni G, Kalantari Z, Albihn A, Björkman C, Bylund H, et al. Healthy Ecosystems for Human and Animal Health: science diplomacy for responsible development in the Arctic: the nordic Centre of Excellence, Clinf.org (Climate-Change effects on the Epidemiology of Infectious diseases and the impacts on Northern Societies). Polar Record. 2021;57:e39.

[19] OIE. Terrestrial Animal Health Code: Glossary [Internet]. 2021. https://www.oie.int/fileadmin/Home/eng/Health_standards/tahc/current/glossaire.pdf

[20] Garritty C, Gartlehner G, Nussbaumer-Streit B, King VJ, Hamel C, Kamel C, et al. Cochrane Rapid Reviews Methods Group offers evidence-informed guidance to conduct rapid reviews. J Clin Epidemiol. 2021;130:13–22. 33068715 10.1016/j.jclinepi.2020.10.007 PMC7557165

[21] LimeSurvey Project Team / Carsten Schmitz. LimeSurvey: An Open Source survey tool [Internet]. 2021. http://www.limesurvey.org

[22] Canadian Animal Health Surveillance System (CAHSS). Reportable & Notifiable Diseases [Internet]. [cited 2022 Oct 8]. https://cahss.ca/cahss-tools/reportable--notifiable-diseases

[23] Canadian Food Inspection Agency. Government of Canada. 2024 [cited 2024 Mar 11]. Reportable diseases: Terrestrial animals. https://inspection.canada.ca/animal-health/terrestrial-animals/diseases/reportable/eng/1303768471142/1303768544412

[24] Public Health Agency of Canada. Government of Canada. 2024 [cited 2024 Mar 11]. Case definitions: Nationally notifiable diseases. https://diseases.canada.ca/notifiable/diseases-list

[25] Dewar M. Nunavik Trichinosis Prevention Program [Internet]. 2020. https://www.makivik.org/article/nunavik-trichinosis-prevention-program/

[26] Institute NR. Nunavut Trichinella Detection Pilot Program [Internet]. https://www.nri.nu.ca/research-programs-and-partnerships

[27] Messier V. La séroprévalence des zoonoses au Nunavik: Surveillance, identification des facteurs de risque et intervention [Internet]. Vol. Maîtrise, Département de médecine sociale et préventive. [Québec]: Université Laval; 2010. http://hdl.handle.net/20.500.11794/22220

[28] Wildlife and Research Department of Environment. Department of Environment, Wildlife and Research: Activity Report [Internet]. Akulivik: Makivik Corporation. 2021 Apr p. 19. (Makivik Annual General Meeting). https://www.makivik.org/wp-content/uploads/2021/05/10.1-2021-04-12_DEWR-AGM-Report.pdf

[29] Canada G of Surveillance of West Nile virus [Internet]. https://www.canada.ca/en/public-health/services/diseases/west-nile-virus/surveillance-west-nile-virus.html

[30] Canadian Wildlife Health Cooperative (CWHC). CWHC-RCSF: Canadian Wildlife Health Cooperative - Réseau canadien pour la santé de la faune. 2023 [cited 2023 Dec 22]. West Nile Virus. https://www.cwhc-rcsf.ca/west_nile_virus.php

[31] Qanuippitaa? National Inuit Health Survey [Internet]. 2021. Available from: https://nationalinuithealthsurvey.ca/.

[32] Ducrocq J, Ndao M, Yansouni CP, Proulx JF, Mondor M, Hamel D, et al. Epidemiology associated with the exposure to Toxoplasma Gondii in Nunavik’s Inuit population using the 2017 Qanuilirpitaa cross-sectional health survey. Zoonoses Public Health. 2021;68:803–14. 34254450 10.1111/zph.12870

[33] Aguilar XF, Leclerc LM, Mavrot F, Olokhaktomiut Hunters & Trappers Committee. An integrative and multi-indicator approach for wildlife health applied to an endangered caribou herd. Sci Rep. 2023;13(1):16524. 10.1038/s41598-023-41689-y PMC10545743 37783688

[34] Benson K, Gareis J, Hammer N, Storr A. 2019 Compendium of Research in the Northwest Territories [Internet]. Inuvik, Northwest Territories: Aurora Research Institute - Department of Environment and Natural Resources; 2019 [cited 2023 Nov 9] p. 148. Report No.: ISSN: 1205–3910. https://nwtresearch.com/sites/default/files/2019_compendium_final_w_covers.pdf

[35] Campbell M. Southampton Island caribou study; 1995.

[36] Campbell M. Qamanirjuaq caribou monitoring program; 2002.

[37] Campbell M. The Kivalliq caribou monitoring program; 2009.

[38] Environment and Climate Change Canada (ECCC). Environment and climate change Canada (ECCC). 2020 [cited 2023 Nov 14]. Caribou in Canada. https://www.canada.ca/en/environment-climate-change/services/species-risk-education-centre/caribou.html

[39] Johnson D, Harms NJ, Larter NC, Elkin BT, Tabel H, Wei GJ. Serum biochemistry, serology, and parasitology of boreal caribou (Rangifer tarandus caribou) in the Northwest territories, Canada. J Wildl Dis. 2010;46(4):1096–107. 20966261 10.7589/0090-3558-46.4.1096

[40] Jonathon Michel EHJ, Gareis K, Benson CO. 2015 compendium of research in the Northwest territories [Internet]. Aurora Research Institute; 2015. p. 162. https://nwtresearch.com/sites/default/files/compendium_2015_web.pdf

[41] Kristi Benson DKR, Davis J, Gareis N, Hammer J, Michel TH. 2017 Compendium of research in the Northwest territories [Internet]. Aurora Research Institute; 2017. p. 178. https://nwtresearch.com/sites/default/files/ari_compendium_2017_web_final.pdf

[42] Kristi Benson JM, Jolie Gareis. 2016 Compendium of research in the Northwest territories [Internet]. Aurora Research Institute; 2016. p. 179. https://nwtresearch.com/sites/default/files/compendium_2016-web.pdf

[43] Kristi Benson JMJ, Gareis N. Hammer. 2018 Compendium of research in the Northwest territories [Internet]. Aurora Research Institute; 2018. p. 169. https://nwtresearch.com/sites/default/files/2018_compendium_-_web_edition1.pdf

[44] Worthington L, Ganton A, Leclerc LM, Davison T, Wilson J, Duclos I. Management Plan for the Dolphin and Union Caribou (Rangifer tarandus groenlandicus x pearyi) in the Northwest Territories and Nunavut 2018 [Internet]. Northwest Territories; 2018 [cited 2023 Nov 10] p. 107. https://www.nwtspeciesatrisk.ca/sites/enr-species-at-risk/files/dolphin_union_caribou_mgmt_plan_2018_final.pdf

[45] Applejohn A. Compendium of Research in the Northwest territories [Internet]. Aurora Research Institute; 2002. p. 117. https://nwtresearch.com/sites/default/files/2002_compendium_0.pdf

[46] Ball MC, Fulton TL, Wilson GA. Genetic Analyses of Wild Bison in Alberta, Canada: implications for recovery and disease management. J Mammal. 2016;97(6):1525–34.

[47] Environment and Climate Change Canada. Recovery Strategy for the Wood Bison (Bison bison athabascae) in Canada [Internet]. Ottawa, Canada: Environment and Climate Change Canada. 2018 [cited 2023 Nov 10] p. 59. (Species at Risk Act Recovery Strategy Series). https://wildlife-species.az.ec.gc.ca/species-risk-registry/virtual_sara/files//plans/Rs-WoodBison-v00-2018Aug-Eng.pdf

[48] Government of Northwest Territories. Slave River Lowlands Bison Management Pla [Internet]. Environment and Natural Resources; 2019 [cited 2023 Nov 9] p. 49. Report No.: 154. https://www.gov.nt.ca/ecc/sites/ecc/files/resources/154_file.pdf

[49] Government of Yukon. Monitoring wildlife health [Internet]. https://yukon.ca/en/wildlife-health#monitoring-wildlife-health

[50] Government of Yukon. Management Plan for the Aishihik Wood Bison (Bison bison athabascae) herd in southwestern Yukon. Whitehorse, Yukon: Environment Yukon; 2012. p. 28.

[51] Harms NJ, Jung TS, Andrew CL, Surujballi OP, VanderKop M, Savic M, et al. Health Status of Reintroduced Wood Bison (Bison Bison Athabascae): assessing the Conservation Value of an isolated Population in Northwestern Canada. J Wildl Dis. 2019;55(1):44–53. 29953313 10.7589/2017-09-235

[52] Heikkila K, Applejohn A. 2004–2005 Compendium of Research in the Northwest Territories [Internet]. Fort Smith: Aurora Research Institute - Department of Environment and Natural Resources; 2008 p. 318. Report No.: ISSN: 1205–3910. https://nwtresearch.com/sites/default/files/2004-05_compendium.pdf

[53] Mercer A. Compendium of Research in the Northwest Territories [Internet]. Yellowknife: Aurora Research Institute - Department of Environment and Natural Resources; 2014 p. 163. Report No.: ISSN: 1205–3910. https://nwtresearch.com/sites/default/files/compendium_2014.pdf

[54] Nishi JS, Dragon DC, Elkin BT, Mitchell J, Ellsworth TR, Hugh-Jones ME. Emergency Response Planning for Anthrax Outbreaks in Bison Herds of Northern Canada. Ann New York Acad Sci. 2002;969:245–50. 10.1111/j.1749-6632.2002.tb04386.x 12381599

[55] Nishi JS, Ellsworth TR, Lee N, Dewar D, Elkin BT, Dragon DC. Northwest Territories. An outbreak of anthrax (Bacillus anthracis) in free-roaming bison in the Northwest Territories, June-July 2006. Canadian Veterinary J = La revue veterinaire canadienne. 2007;48:37–8. PMC1716754 17310621

[56] Walker V. 2000 Compendium of research in the Northwest territories [Internet]. Aurora Research Institute; 2000. p. 108. https://nwtresearch.com/sites/default/files/2000_compendium.pdf

[57] Yukon G of Yukon research compendium [Internet]. https://yukon.ca/en/wildlife-health#monitoring-wildlife-health

[58] Fisheries Joint Management Committee. Beaufort Sea Beluga Management Plan. 4th amended Printing. Northwest Territories: Inuvik; 2013. p. 56.

[59] Lisa Loseto MN. Sonja Ostertag. Beluga Sampling on Hendrickson Island: Summary Report - Field Season 2010; 2010.

[60] Ostertag SK, Loseto LL, Snow K, Lam J, Hynes K, Gillman DV. That’s how we know they’re healthy: the inclusion of traditional ecological knowledge in beluga health monitoring in the Inuvialuit Settlement Region. Arctic Sci. 2018;4:292–320.

[61] Coxon A. Polar Bear Laboratory - Harvest Program. 2008.

[62] Environment and Climate Change Canada (ECCC). Polar Bear (Ursus maritimus): management plan progress report, March 2023 [Internet]. 2023 [cited 2023 Nov 16]. https://www.canada.ca/en/environment-climate-change/services/species-risk-public-registry/report-progress-recovery-document/polar-bear-management-plan-progress-report-march-2023.html

[63] Government of Nunavut. Nunavut Polar Bear Co-management plan [Internet]. Government of Nunavut; 2019 [cited 2023 Nov 16]. https://gov.nu.ca/sites/default/files/nwmb_approved_polar_bear_comanagement_plan_sept_2019_eng.pdf

[64] Joint Secretariat. Inuvialuit settlement region polar bear joint management plan [Internet]. Inuvialuit Settlement Region; 2017 [cited 2023 Nov 16]. https://www.nwtspeciesatrisk.ca/sites/enr-species-at-risk/files/isr_polar_bear_joint_management_plan_2017_final.pdf

[65] Polar Bear Management Plan for Québec, the Eeyou Marine Region and the Nunavik Marine Region [Internet]. 2022 [cited 2023 Nov 16]. https://www.emrwb.ca/wp-content/uploads/2023/04/202209-Quebec_EMR_NMR-Polar-Bear-Management-Plan_EN_FINAL.pdf

[66] Mercer A, Allooloo S, Dutton J. Compendium of research in the Northwest territories [Internet]. Fort Smith: Aurora Research Institute - Department of Environment and Natural Resources; 2013 p. 157. Report No.: ISSN: 1205–3910. http://library.assembly.gov.nt.ca/2013/ARI/a354815_2013.pdf

[67] Mercer A, Applejohn A. Compendium of research in the Northwest territories [Internet]. Fort Smith: Aurora Research Institute - Department of Environment and Natural Resources; 2007 p. 133. Report No.: ISSN: 1205–3910. https://nwtresearch.com/sites/default/files/2007_compendium.pdf

[68] Mercer A, Seccombe-Hett P. Compendium of research in the Northwest Territories [Internet]. Fort Smith: Aurora Research Institute - Department of Environment and Natural Resources; 2008 p. 137. Report No.: ISSN: 1205–3910. https://nwtresearch.com/sites/default/files/2008_compendium.pdf

[69] Michel J, Gareis J, Benson K, Owen C, Hille E. 2015 Compendium of Research in the Northwest Territories [Internet]. Yellowknife: Aurora Research Institute - Department of Environment and Natural Resources; 2015 p. 162. Report No.: ISSN: 1205–3910. https://nwtresearch.com/sites/default/files/compendium_2015_web.pdf

[70] Trimble A, Hille E, Gareis J, Michel J, Hammer N, Seccombe-Hett P et al. 2009–2010 Compendium of Research in the Northwest Territories [Internet]. Fort Smith: Aurora Research Institute - Department of Environment and Natural Resources; 2012 p. 337. Report No.: ISSN: 1205–3910. https://nwtresearch.com/sites/default/files/2009-2010-compendium-of-research-in-the-nwt.pdf

[71] Di Francesco J, Kwong G, Deardon R, Checkley S, Mastromonaco G, Mavrot F et al. Qiviut cortisol is associated with metrics of health and other intrinsic and extrinsic factors in wild muskoxen (Ovibos moschatus). Conserv Physiol. 2022;10(1). 10.1093/conphys/coab103 PMC9040286 35492408

[72] Leclerc LM. Muskox Disease Monitoring, 2013–2014. 2013.

[73] Nunavut Research Institute (NRI). 2020 Compendium of licensed research in nunavut [Internet]. Nunavut Research Institute; 2021. p. 92. https://www.nri.nu.ca/sites/default/files/documents/2020_Compendium_Final_0.pdf

[74] The Wildlife Management Advisory Council (North Slope). Framework for the management of Yukon North Slope Muskox [Internet]. The Wildlife Management Advisory Council (Northwest Territories); 2017 [cited 2023 Nov 16]. https://wmacns.ca/resources/framework-management-north-slope-muskox/

[75] Tomaselli M. Musk ox health & resilience: musk ox surveillance on Victoria Island to support food security, food safety, public health & musk ox health; 2015.

[76] Tomaselli M, Dalton C, Duignan PJ, Kutz S, van der Meer F, Kafle P, et al. Contagious Ecthyma, Rangiferine Brucellosis, and Lungworm Infection in a Muskox (Ovibos moschatus) from the Canadian Arctic, 2014. J Wildl Dis. 2016;52:719–24. 27285415 10.7589/2015-12-327

[77] Tomaselli M, Kutz S, Gerlach C, Checkley S. Local knowledge to enhance wildlife population health surveillance: conserving muskoxen and caribou in the Canadian Arctic. Biological Conservation. 2018;217:337–48.

[78] Tomaselli M, Elkin B, Kutz S, Harms NJ, Nymo HI, Davison T, et al. A Transdisciplinary approach to brucella in muskoxen of the western Canadian Arctic 1989–2016. EcoHealth. 2019;16(3):488–501. 31414318 10.1007/s10393-019-01433-3 PMC6858907

[79] Applejohn A. Compendium of Research in the Northwest Territories [Internet]. Fort Smith: Aurora Research Institute - Department of Environment and Natural Resources; 2001 p. 109. Report No.: ISSN: 1205–3910. https://nwtresearch.com/sites/default/files/2001_compendium.pdf

[80] Applejohn A. Compendium of research in the northwest territories [Internet]. Fort Smith: Aurora Research Institute - Department of Environment and Natural Resources; 2002 p. 117. Report No.: ISSN: 1205–3910. https://nwtresearch.com/sites/default/files/2002_compendium_0.pdf

[81] Applejohn A. Compendium of Research in the Northwest Territories [Internet]. Fort Smith: Aurora Research Institute - Department of Environment and Natural Resources; 2003 p. 136. Report No.: ISSN: 1205–3910. https://nwtresearch.com/sites/default/files/2003_compendium.pdf

[82] Bouchard É, Sharma R, Hernández-Ortiz A, Buhler K, Al-Adhami B, Su C, et al. Are foxes (Vulpes spp.) good sentinel species for Toxoplasma Gondii in northern Canada? Parasit Vectors. 2022;15(1):115. 35365191 10.1186/s13071-022-05229-3 PMC8972674

[83] Campbell M. Wildlife disease monitoring in the Kivalliq. 2000.

[84] Canadian Wildlife Health Cooperative (CWHC). CWHC Surveillance & Response [Internet]. http://www.cwhc-rcsf.ca/surveillance_and_response.php

[85] Larter NC, Forbes LB, Elkin BT, Allaire DG. Prevalence of Trichinella spp. in Black bears, Grizzly bears, and wolves in the Dehcho Region, Northwest Territories, Canada, including the First Report of T. Nativa in a Grizzly Bear from Canada. J Wildl Dis. 2011;47:745–9. 21719845 10.7589/0090-3558-47.3.745

[86] Larter NC, Elkin BT, Forbes LB, Wagner B, Allaire DG. Trichinella Surveillance in Black Bears (Ursus americanus) from the Dehcho Region, Northwest Territories, Canada, 2002-15. J Wildl Dis. 2017;53:405–7. 28094606 10.7589/2016-06-135

[87] Mercer A, Trimble A. Compendium of Research in the Northwest Territories [Internet]. Fort Smith: Aurora Research Institute - Department of Environment and Natural Resources; 2006 p. 127. Report No.: ISSN: 1205–3910. https://nwtresearch.com/sites/default/files/2006_compendium.pdf

[88] Nunavut Wildlife Management Board. Community-based wildlife monitoring network [Internet]. https://www.nwmb.com/en/cbmn

[89] Sharma R, Parker S, Elkin B, Mulders R, Branigan M, Pongracz J, et al. Risk factors and prevalence of antibodies for Toxoplasma Gondii in diaphragmatic fluid in wolverines (Gulo gulo) from the Northwest territories, Canada. Food Waterborne Parasitol. 2019;15:8. 10.1016/j.fawpar.2019.e00056 PMC7034056 32095625

[90] Stephen C. The Canadian Wildlife Health Cooperative: Addressing wildlife health challenges in the 21st century. The Canadian Vet J = La revue veterinaire canadienne. 2015;56:925–7. PMC4535505 26346834

[91] United Kingdom Research and Innovation. CINUK. 2023 [cited 2023 Nov 9]. ArcticEID - community-based wildlife surveillance. https://www.cinuk.org/projects/arcticeid/

[92] Watson SE, Hailer F, Lecomte N, Kafle P, Sharma R, Jenkins EJ et al. Parasites of an Arctic scavenger; the wolverine (Gulo gulo). Int J Parasitology-Parasites Wildlife. 2020;13:178–85. 10.1016/j.ijppaw.2020.10.004 PMC7591336 33134077

[93] Wayne Condon JGN, Hammer E, Hille A, Mercer J, Michel SR, Pippa Seccombe H. 2011–2012 Compendium of Research in the Northwest Territories [Internet]. Fort Smith: Aurora Research Institute; 2014. p. 230. Report No.: ISSN: 1205–3910. https://nwtresearch.com/sites/default/files/compendium_2011-2012.pdf

[94] Wobeser G, Campbell GD, Dallaire A, McBurney S. Tularemia, plague, yersiniosis, and Tyzzer’s disease in wild rodents and lagomorphs in Canada: a review. Can Vet J. 2009;50(12):1251–6. 20190973 PMC2777287

[95] Brook RK, Kutz SJ, Veitch AM, Popko RA, Elkin BT, Guthrie G. Fostering Community-Based Wildlife Health Monitoring and Research in the Canadian North. EcoHealth. 2009;6(2):266–78. 19953294 10.1007/s10393-009-0256-7

[96] Kutz Research Group. Muskox and Caribou Health Monitoring Program 2021 [Internet]. University of Calgary; 2021 [cited 2023 Nov 9]. https://drive.google.com/file/d/1WlmpIHOOcU-eigGGKeQdWXaIMyZxxyPm/view?fbclid=IwAR0QGBTM-Z3LzA5f5fESuWxOGl8xq2Qi2P-cCgIu2fW7-Go6cS2-yMju9R8%26usp=embed_facebook

[97] Kutz S, D J, Cuyler C, Elkin B, Gunn A, Kolpashikov L, Russell D, White RG. Standardized monitoring of Rangifer health during International Polar Year. Rangifer. 2013;33(2):91–114.

[98] Kutz SJ, Hoberg EP, Nagy J, Polley L, Elkin B. Emerging parasitic infections in arctic ungulates. Integrative Comparative Biol. 2004;44:109–18. 10.1093/icb/44.2.109 21680491

[99] dos Ribeiro S, van de Burgwal C, Regeer LHM. Overcoming challenges for designing and implementing the One Health approach: a systematic review of the literature. One Health. 2019;7:100085. 31016220 10.1016/j.onehlt.2019.100085 PMC6475629

[100] Clow KM, Leighton PA, Pearl DL, Jardine CM. A framework for adaptive surveillance of emerging tick-borne zoonoses. One Health. 2019;7:100083. 30809583 10.1016/j.onehlt.2019.100083 PMC6376153

[101] Jenkins EJ, Schurer JM, Gesy KM. Old problems on a new playing field: Helminth zoonoses transmitted among dogs, wildlife, and people in a changing Northern climate. Vet Parasitol. 2011;182:54–69. 21802208 10.1016/j.vetpar.2011.07.015

[102] Sawatzky A, Cunsolo A, Jones-Bitton A, Middleton J, Harper SL. Responding to Climate and Environmental Change Impacts on Human Health via Integrated Surveillance in the Circumpolar North: a systematic Realist Review. Int J Environ Res Public Health. 2018;15:2706. 30513697 10.3390/ijerph15122706 PMC6313572

[103] Awuor L, Meldrum R, Liberda EN. Prospects of leveraging an existing mosquito-borne disease surveillance system to monitor other emerging mosquito-borne diseases: a systematic review of West Nile Virus surveillance in Canada (2000–2016). Environ Health Rev. 2019;62(3):82–91.

[104] Davis K, Ford D, Quinn J, Research Team CIHACC, Harper L. S. From participatory engagement to co-production: modelling climate-sensitive processes in the Arctic. Arctic Sci. 2021;7:699–722.

[105] Van Bavel B, Ford LB, Harper SL, Ford J, Elsey H, Lwasa S, et al. Contributions of scale: what we stand to gain from indigenous and local inclusion in climate and health monitoring and surveillance systems. Environ Res Lett. 2020;15(8):22.

[106] Keatts LO, Robards M, Olson SH, Hueffer K, Insley SJ, Joly DO et al. Implications of Zoonoses From Hunting and Use of Wildlife in North American Arctic and Boreal Biomes: pandemic potential, monitoring, and mitigation. Front Public Health [Internet]; 2021 [cited 2022 Oct 8];9. https://www.frontiersin.org/articles/10.3389/fpubh.2021.627654 10.3389/fpubh.2021.627654 PMC8131663 34026707

[107] Ford JD, Willox AC, Chatwood S, Furgal C, Harper S, Mauro I, et al. Adapting to the effects of climate change on inuit health. Am J Public Health. 2014;104(Suppl 3):e9–17. 24754615 10.2105/AJPH.2013.301724 PMC4035894

[108] Furgal C, Seguin J. Climate change, health, and vulnerability in Canadian Northern aboriginal communities. Environ Health Perspect. 2006;114(12):1964–70. 17185292 10.1289/ehp.8433 PMC1764172

[109] Peacock J, Mavrot S, Tomaselli F, Hanke M, Fenton A, Nathoo H. Linking co-monitoring to co-management: bringing together local, traditional, and scientific knowledge in a wildlife status assessment framework. Arct Sci. 2020;6(3):247–66.

[110] Johnson N, Behe C, Danielsen F, Krümmel EM, Nickels S, Pulsifer PL. Community-Based Monitoring and Indigenous Knowledge in a Changing Arctic: A Review for the Sustaining Arctic Observing Networks. Final report to Sustaining Arctic Observing Networks. [Internet]. Ottawa, ON: Inuit Circumpolar Council; 2016 p. 74. https://www.inuitcircumpolar.com/project/community-based-monitoring-and-indigenous-knowledge-in-a-changing-arctic-a-review-for-the-sustaining-arctic-observing-networks%E2%80%8B/

[111] Canada in a Changing Climate. Canada in a Changing Climate; 2023 [cited 2023 Nov 14]. Map of Adaptation Actions. https://changingclimate.ca/map/

[112] Wekʼèezhìi Renewable Resources Board. Northern Caribou Canada. [cited 2023 Nov 14]. Northern Caribou Canada. https://www.northerncaribou.ca/

[113] Parkinson AJ, Bruce MG, Zulz T. International Circumpolar Surveillance, an Arctic Network for the Surveillance of Infectious diseases. Emerging Infectious Dis J. 2008;14:18. 10.3201/eid1401.070717 PMC2600151 18258072

[114] Bruce M, Zulz T, Koch A. Surveillance of infectious diseases in the Arctic. Public Health. 2016;137:5–12. 27473191 10.1016/j.puhe.2016.06.014

[115] Bourgeois AC, Zulz T, Soborg B, Koch A. Descriptive review of Tuberculosis Surveillance systems across the circumpolar regions. Int J Circumpolar Health. 2016;75:30322. 27121178 10.3402/ijch.v75.30322 PMC4848390

[116] Bourgeois AC, Zulz T, Bruce MG, Stenz F, Koch A, Parkinson A, et al. Tuberculosis in the circumpolar region, 2006–2012. Int J Tuberculosis Lung Disease. 2018;22(6):641–8. 10.5588/ijtld.17.0525 29862948

[117] Finlayson-Trick E, Barker B, Manji S, Harper SL, Yansouni CP, Goldfarb DM. Climate Change and Enteric Infections in the Canadian Arctic: do we know what’s on the Horizon? Gastrointest Disord. 2021;3(3):113–26.

[118] Jenkins EJ, Castrodale LJ, de Rosemond SJC, Dixon BR, Elmore SA, Gesy KM et al. Tradition and Transition: Parasitic Zoonoses of People and Animals in Alaska, Northern Canada, and Greenland. In: Rollinson D, editor. Advances in parasitology, Vol. 82 [Internet]. San Diego: Elsevier Academic Press Inc; 2013. pp. 33–204. 10.1016/B978-0-12-407706-5.00002-2 10.1016/B978-0-12-407706-5.00002-2 23548085

[119] Kutz S, Bollinger T, Branigan M, Checkley S, Davison T, Dumond M, et al. Erysipelothrix rhusiopathiae associated with recent widespread muskox mortalities in the Canadian Arctic. Can Vet J. 2015;56(6):560–3. 26028673 PMC4431149

[120] Buhler KJ, Dibernardo A, Pilfold NW, Harms NJ, Fenton H, Carriere S, et al. Widespread exposure to Mosquitoborne California Serogroup Viruses in Caribou, Arctic Fox, Red Fox, and Polar bears, Canada. Emerg Infect Dis. 2023;29(1):54–63. 36573538 10.3201/eid2901.220154 PMC9796188

[121] Villeneuve CA, Buhler KJ, Iranpour M, Avard E, Dibernardo A, Fenton H et al. New records of California serogroup viruses in Aedes mosquitoes and first detection in simulioidae flies from Northern Canada and Alaska. Polar Biol. 2021;44:1911–5.

[122] Armstrong T, Rogers G, Rowley G. The Circumpolar North: a political and economic geography of the Arctic and Sub-arctic. Taylor & Francis; 2023. p. 336.

[123] Kulkarni T, Watkins JM, Nickels S, Lemmen DS. Canadian International Polar Year (2007–2008): an introduction. Clim Change. 2012;115(1):1–11.

[124] Proulx JF, MacLean JD, Gyorkos TW, Leclair D, Richter AK, Serhir B et al. Novel prevention program for trichinellosis in inuit communities. Clinical Infectious Dis. 2002;34:1508–14. 10.1086/340342 12015698

[125] Larrat S, Simard M, Lair S, Bélanger D, Proulx JF. From science to action and from action to science: the Nunavik trichinellosis prevention program. Int J Circumpolar Health. 2012;71:18595. 22789519 10.3402/ijch.v71i0.18595 PMC3417525

[126] Martinez-Levasseur LM, Simard M, Furgal CM, Burness G, Bertrand P, Suppa S et al. Towards a better understanding of the benefits and risks of country food consumption using the case of walruses in Nunavik (Northern Quebec, Canada). Sci Total Env. 2020;719:137307. 10.1016/j.scitotenv.2020.137307 32143094

[127] Provincial Health Services Authority. BC Centre for Disease Control. 2023 [cited 2023 Dec 14]. West Nile Virus (WNV) - Surveillance. http://www.bccdc.ca/health-info/diseases-conditions/west-nile-virus-wnv/surveillance

[128] Lévesque B, Lavoie É, Proulx J, Libman MD, Grant J, Gingras S et al. Evaluation of the Toxoplasmosis Screening Program Among Pregnant Nunavik (Canada) Women Between 1994–2003 [Internet]. Vol. 18; 2007. pp. S32–3. https://journals.lww.com/epidem/Fulltext/2007/09001/Evaluation_of_the_Toxoplasmosis_Screening_Program.94.aspx

[129] Lavoie É, Lévesque B, Proulx JF, Grant J, Ndassebe AD, Gingras S, et al. Évaluation Du Programme De dépistage de la toxoplasmose chez les femmes enceintes du Nunavik, 1994–2003. Can J Public Health. 2008;99:397–400. 19009924 10.1007/BF03405249 PMC6976170

