# Supplementary material for: A call for a coherent One Health strategy for the surveillance of climate-sensitive infectious diseases in the Canadian Arctic and subarctic regions
Source: One Health Outlook. 2024 Dec 1;6:25. doi: 10.1186/s42522-024-00117-5 (PMC11608495; doi:10.1186/s42522-024-00117-5)
Supplement: Supplementary file 1 — Supplementary Material 1 [file 42522_2024_117_MOESM1_ESM.pdf]

Appendix 1. Keywords used – Boolean operators

Boolean operators could vary depending on the database used. Different combinations were carried out to ensure that we had all the records to meet the objectives of this study.

(surveillance? OR track* OR monitor* OR activit* OR network? OR project? OR program?)

AND

(climate OR "climate-sensitive" OR bacteria OR disease? OR illness OR infection? OR pathogen? OR virus* OR "bacillus anthracis" OR Anthrax OR Borreli* OR "Lyme disease" OR "Brucell*" OR "Clostridium botulinum" OR botulism OR "coxiella burnetti" OR "Q Fever" OR "Francisella tularensis" OR tularemia OR "Leptospir*" OR Cryptosporidi* OR Echinococc* OR giardia* OR toxoplasma* OR trichinell* OR hantavirus* OR rabies OR "tick-borne encephalitis virus*" OR "West Nile virus")

AND

("Canadian Arctic" OR "Canadian subarctic" OR "circumpolar" OR "Labrador" OR "Northwest Territories" OR Nunavut OR Yukon OR Inuvialuit OR Nunatsiavut OR Nunatukavut OR Nunavik)

AND

(surveillance? OR track* OR monitor* OR activit* OR network? OR project? OR program?)

AND / OR

("One Health" OR Ecohealth)
