# Supplementary material for: A call for a coherent One Health strategy for the surveillance of climate-sensitive infectious diseases in the Canadian Arctic and subarctic regions
Source: One Health Outlook. 2024 Dec 1;6:25. doi: 10.1186/s42522-024-00117-5 (PMC11608495; doi:10.1186/s42522-024-00117-5)
Supplement: Supplementary file 2 — Supplementary Material 2 [file 42522_2024_117_MOESM2_ESM.pdf]

Appendix 2. Key stakeholder questionnaire

The questionnaire was composed of five questions to collect additional information about ongoing or past surveillance activities or programs.

1) Is the [*Name of the program/network*] currently involved in any ongoing surveillance **and/or monitoring** activities for infectious or environmental diseases in humans or animals?

   -> Provide examples of animal, humans and combined surveillance projects.

2) If yes, which infectious or environmental diseases or pathogens are the outcome of interest of this surveillance?
3) Is there further information on these surveillance activities, and who would be the main point of contact?
4) Do you any other key informant we should contact about our project?
5) Do you know of other surveillance and/or monitoring activities taking place in your area, and who would be the best person to contact for more information?
