# Supplementary material for: A call for a coherent One Health strategy for the surveillance of climate-sensitive infectious diseases in the Canadian Arctic and subarctic regions
Source: One Health Outlook. 2024 Dec 1;6:25. doi: 10.1186/s42522-024-00117-5 (PMC11608495; doi:10.1186/s42522-024-00117-5)
Supplement: Supplementary file 3 — Supplementary Material 3 [file 42522_2024_117_MOESM3_ESM.pdf]

Appendix 3

Table S1. Information on surveillance programs extracted from included documentation

Variable	Predefined values	Definition	Examples of values from the data extraction
Diseases	Anthrax	*Bacillus anthracis*	Anthrax; Bacillus anthracis
	Botulism	*Clostridium botulinum*	Botulism; Clostridium botulinum
	Brucellosis	*Brucella sp.*	Brucellosis; Brucella sp.
	Leptospirosis	*Leptospira sp.*	Leptospirosis; Leptospira sp.
	Tularemia	*Francisella tularensis*	Tularemia; Francisella tularensis
	Lyme disease	*Borrelia burgdorferi*, Lyme encephalitis	Lyme disease; Borrelia burgdorferi, Lyme encephalitis
	Q fever	*Coxiella burnetti*	Q fever; Coxiella burnetti
	Hantavirus pulmonary syndrome	*Hantavirus*	Hantavirus pulmonary syndrome; Hantavirus
	Rabies	Rabies, rhabdovirus	Rabies; Rabies, rhabdovirus
	Tick-borne encephalitis	TBE, tick-borne encephalitis virus	Tick-borne encephalitis; TBE, tick-borne encephalitis virus
	West Nile virus	West Nile virus, WNV	West Nile fever; West Nile virus, WNV, West Nile virus encephalitis
	Cryptosporidiosis	*Cryptosporidium sp.*	Cryptosporidiosis; Cryptosporidium sp.
	Echinococcosis	*Echinococcus sp.*	Echinococcosis; Echinococcus sp.
	Giardiasis	*Giardia sp.*	Giardiasis; Giardia sp.
	Toxoplasmosis	*Toxoplasma sp.*	Toxoplasmosis; Toxoplasma sp.
	Trichinellosis	*Trichinella sp.*	Trichinellosis; Trichinella sp.
	Unknown (probable CSIDs)	Diseases monitored are not explicitly named.	
Type of data collection	Opportunistic	Disease(s) are identified through other research or monitoring activities.	
	Passive	Regular, ongoing reporting of diseases by the appropriate authority.	
	Active	Efforts are carried to investigate the presence of the disease(s) in the population(s) of interest.	

Geographic Area	Canada	Country-wide	Canada, National, Federal
	NWT	Northwest Territories	Northwest Territories, Inuvialuit Settlement Region, Sahtú Settlement Area, Mackenzie, Slave River Lowlands, Nahanni, Bison Control Area, Liard, Wood Buffalo National Park, Hendrickson Island, Tuktoyaktuk, Beaufort Sea, Inuvik, Paulatuk
	YT	Yukon	Yukon
	NU	Nunavut	Nunavut, Cambridge Bay, Kugaaruk, Baker Lake, Taloyoak, Clyde River, Arviat, Sanikiluaq, Whale Cove, Rankin Inlet, Southampton Island
	NFL	Newfoundland and Labrador	Newfoundland and Labrador, Nunatsiavut
	NK	Nunavik	Québec, Nunavik
Stakeholders	Federal	Federal government, its departments, and agencies	Environment Canada, Fisheries and Oceans Canada, Public Health Agency of Canada, Parks Canada, Canadian Wildlife Service, Polar Knowledge Canada
	Provincial/Territorial	Provincial or Territorial Goverments, its departments, and agencies	Government of Newfoundland and Labrador, Gouvernement du Québec, Yukon Government, Government of Nunavut, Government of Northwest Territories<br>Nunavut Dep. of Sustainable Development, Nunavut Dep. Of Environment, Ministère de la Santé et des Services Sociaux, Ministère de l'Agriculture, des Pêcheries et de l'Alimentation, Institut National de Santé Publique du Québec, Ministère des Forêts, de la Faune et des Parcs, NWT Dep. Of Health and Social Services, NWT Dep. of Environmental and Natural Resources, Yukon Dept. of Animal Health Unit
	Regional/Local	Indigenous, regional, or local governments and its branches	Nunavik Regional Board of Health and Social Services, Inuit Tapiriit Kanatami, Inuvialuit Regional Corporation, Nunavut Tuungavik Incorporated, Makivik Corporation, Nunatsiavut Government, Nunavik Research Centre, Nunavut Research Institute, Kativik Regional Government

	Special Interest Groups	Local Hunter and Trapper's Associations, Wildlife Management Boards	Nunavut Wildlife Management Board, Local Hunter and Trapper Organizations, Beverly and Qamanirjuaq Caribou Management Board, Kivalliq Wildlife Board, Inuvialuit Hunter and Trappers Committee
	NGO	Nongovernmental organization, Non-profit organization	Wildlife Conservation Society Canada, Canadian Wildlife Health Cooperative, Canadian Wildlife Federation, Centre for Coastal Health
	Academia	Researchers affiliated with academia and universities.	University of Calgary, University of Saskatchewan, University of Prince Edwards Island, University of Montreal, University of Northern British Columbia
Sectors under surveillance	Domestic animal health	All activities involving the sampling of domestic animals.	Domestic animals, horse
	Human health	All activities involving the sampling of humans.	Humans
	Wildlife and environmental health	All activities involving the sampling of wildlife and/or environmental parameters.	Barren-ground caribou, boreal caribou, wood bison, muskox, wolf, muskrat, fox, wolverine, black bear, grizzly bear, wildlife, fish, birds, mammals, reptiles, amphibians, mosquitoes, beluga
	Plant health	All activities involving the sampling of plants.	No data.
	Food safety	All activities involving the sampling of meat from wildlife or domestic animals harvested for food consumption	Ringed seal, bearded seal, beluga, muskox, whale, caribou, fish, grizzly bear, black bear, ptarmigan, hare, polar bear, walrus
Indigenous Peoples involvement	Lead	Indigenous Peoples were involved as leaders of the surveillance program.	Inuit, First Nation and/or Metis organization are at the core of the program.
	Collaborator	Indigenous Peoples were involved as collaborators in the surveillance program.	Champagne and Aishihik First Nations, Sahtú Dene and Metis, Participating Inuit communities

	Unclear	Indigenous Peoples were involved at any level in the surveillance program, but no specific nation or community was mentioned.
	None	Indigenous Peoples were not involved, involvement is unknown or not disclosed.
Dissemination of information	Grey litterature	Grey literature review: some information on this surveillance program was obtained in the mentioned sources.
	Scientific litterature	Scientific literature review: some information on this surveillance program was obtained in the mentioned sources.
	Consultation with key stakeholders	Online survey: some information on this surveillance program was obtained in the mentioned sources.
